# Intra-Cranial Arterial Calcifications in Hemodialysis Patients

**DOI:** 10.3390/medicina59101706

**Published:** 2023-09-24

**Authors:** Feda Fanadka, Ilan Rozenberg, Naomi Nacasch, Yael Einbinder, Sydney Benchetrit, Ori Wand, Tammy Hod, Keren Cohen-Hagai

**Affiliations:** 1 Department of Radiology, Meir Medical Center, Kfar Saba 4428164, Israel; feda.fanadka@clalit.org.il; 2 Department of Nephrology and Hypertension, Meir Medical Center, Kfar Saba 4428164, Israel; 3 Faculty of Medicine, Tel Aviv University, Tel Aviv 6997801, Israel; 4 Pulmonary Division, Barzilai Medical Center, Ashkelon 7830604, Israel; 5 Department of Nephrology and Hypertension, Sheba Medical Center, Ramat Gan 5262000, Israel

**Keywords:** arterial calcification, hemodialysis, end stage kidney disease, atherosclerosis

## Abstract

*Background and objectives*: Vascular calcification is an integral part of atherosclerosis and has been reported to be an independent risk factor for cardiovascular diSsease. Intra Cranial Arterial Calcifications (ICAC) in maintenance hemodialysis (MHD) is highly prevalent. *Materials and Methods*: The aim of this retrospective study was to assess the predictors and outcomes of ICAC in MHD patients compared to a control group without kidney disease. A blinded neuroradiologist graded ICAC in brain imaging (computerized tomography) of MHD patients. Age- and sex-matched patients with normal kidney function served as the control group. *Results*: A total of 280 patients were included in the cohort; 140 of them were MHD patients with a mean ICAC score of 2.3 ± 0.2 versus a mean ICAC score of 1.4 ± 0.2 in the control group (*p* < 0.01). More than 90% of hemodialysis patients in our study had some degree of ICAC. Lower albumin and higher phosphorus and CRP levels were associated with increased ICACs. The multivariate analysis model for predictors of 1-year mortality demonstrated an increased odds ratio for mortality as the ICAC score increased. *Conclusions:* ICAC is very prevalent among MHD patients and results not simply from passive deposition of calcium and phosphate but rather from complex and active processes involving inflammation and structural changes in blood vessels. ICAC independently predicted all-cause mortality and may help with risk stratification of this high-risk population.

## 1. Introduction

Abnormal structural neuroimaging is commonly seen among patients with chronic kidney disease (CKD) and is even more common among patients with end-stage kidney disease (ESKD) on hemodialysis (HD). Several studies have found structural neuroimaging abnormalities such as cerebral atrophy, white matter hyperintensity, small and large vessel infarcts, and intracranial arterial calcifications. The prevalence of all these abnormalities is higher among HD patients than it is among patients without kidney disease [1,2,3,4].

Intracranial arterial calcifications (ICAC) are a common finding in neuroimaging of HD patients [5]. Arterial calcification is an integral feature of active atherosclerosis, occurring in up to 90% of atheromatous lesions. It may be used as a non-invasive marker of atherosclerosis because calcium deposits can be easily detected on non-contrast CT. A strong correlation has been demonstrated between coronary calcifications and coronary plaques. Coronary artery calcification score is a sensitive marker for predicting acute coronary disease [6,7,8].

ICAC is also considered a relatively sensitive and specific marker of intracranial atherosclerosis in the general population. Aging, traditional cardiovascular risk factors, and CKD have been associated with ICAC. Its presence and severity reflect intracranial atherosclerosis, increased risk of ischemic stroke, vascular events, and death in the general population [6,7,8].

ICAC in HD patients has a prevalence ranging from 76% to 95%, and its severity is correlated with age, hemodialysis vintage, and serum phosphate levels [5,9]. The association between atherosclerosis and calcification has been established in CKD patients, as well as the resultant cardiovascular disease.

Jablonski and Chonchol [10] reviewed the pathophysiology of vascular calcifications in patients with ESRD, which is extensively deposited in the media layer of the vasculature and is associated with increased arterial stiffness that results in increased systolic blood pressure (SBP), reduced diastolic blood pressure (DBP) and consequently, increased pulse pressure (PP). These changes, with their associated increased cardiac afterload, compromise coronary artery perfusion during diastole, and subsequent left ventricular remodeling and hypertrophy are commonly seen. Simultaneously, coronary plaque increases the risk of myocardial infarction and heart failure, and thus, cardiovascular morbidity and mortality [10].

### Aim

The current study assessed the predictors of intracranial arterial calcifications in hemodialysis patients compared to a control group without kidney disease. We also assessed the correlation between ICAC and overall mortality among HD patients.

## 2. Materials and Methods

### 2.1. Study Design

This retrospective case–control cohort study included chronic HD patients who had undergone brain non-contrast computerized tomography (CT) from 1 January 2015 until 31 December 2017. Brain imaging was performed as part of the routine evaluation and management of the patients (e.g., after a fall or head trauma).

### 2.2. Calcification Scoring

A neuroradiologist who was blinded to the clinical and laboratory information of the study population performed repeated and directed radiologic assessment of brain CTs and scored the intracranial arterial calcification. Assessment of thickness of the calcifications in the wall of both common carotid arteries using a score from 0 to 4 as follows [11]: 0—No calcifications, 1—Calcification 1 mm thick or stippled, 2—Calcification 2 mm thick, thin continuous, or thick discontinuous, 3—Calcification 3 mm thick, or thick continuous, 4—Calcification of >3 mm thick or double tracts. For analysis, we have categorized those ICAC results into three categories: patients with a score of 0 were classified as low ICAC, scores 1 and 2 were categorized as medium ICAC and scores of 3 and 4 were grouped as high ICAC.

### 2.3. Study Population and Data Collection

Chronic HD was defined as a minimum of 3 months of dialysis. Most of the dialysis patients were treated using conventional HD schedules (thrice-weekly sessions, 4 hours each) with high-flux membranes. Hypertension in dialysis patients was determined based on a documented diagnosis in the patient’s medical record or prescription of antihypertensive medication. Blood pressure measurements were assessed before, during, and after mid-week dialysis sessions and were published and described previously [12]. We included all chronic HD patients who had undergone non-contrast brain CT during the study period. Follow-up for each participant lasted until 31 December 2017 or until the subject died.

Patients with normal kidney function who had undergone brain CT during a visit to the Emergency Department at Meir Medical Center during the study period were matched for age and gender to the HD group and served as the control group. Relevant data were abstracted from the electronic medical records of the HD and control patients. The reason for performing the imaging was not available for all patients and was not adjusted for between the groups. Laboratory data were also collected from the electronic medical records; for HD patients, we collected pre-dialysis laboratory results performed up to 2 weeks prior to the CT, and for controls, we collected results from tests performed on the day of CT.

### 2.4. Ethical Considerations

The study was approved by the local Institutional Ethics Committee in keeping with the principles of the Declaration of Helsinki. Data were collected anonymously from the electronic medical records without active patient participation.

### 2.5. Statistical Analysis

Data are presented as numbers and percentages for nominal parameters and as means and standard deviations for continuous parameters. Variables between study groups were compared using ANOVA *t*-test, or chi-square test, according to the scale of measured variables. Correlations were assessed using Spearman and Pearson coefficients of correlation.

A multivariate regression analysis model was constructed to assess 1-year mortality. Model building was performed on the basis of univariate testing and included ICAC as well as other variables, which demonstrated a significant univariate correlation with mortality. Kaplan–Meier survival curves were used to compare mortality over time between groups. *p* values < 0.05 were considered statistically significant. Data were analyzed with SPSS Version 25 (IBM Corporation, Armonk, NY, USA).

## 3. Results

A total of 280 patients were included in this study: 140 in the dialysis group and 140 in the matched control group. The mean age of the study cohort was 71 ± 12 years, and 170 (60.7%) were men. Dialysis patients had significantly more traditional cardiovascular risk factors, such as diagnosis of hypertension and diabetes mellitus, and a higher prevalence of cardiovascular disease (peripheral vascular disease, ischemic heart disease, congestive heart failure) than the control group did. Basic demographic data and comorbidities according to ICAC groups are shown in Table 1. The prevalence of hypertension, diabetes mellitus, and atherosclerosis (coronary heart disease and peripheral vascular disease) was higher as the ICAC was higher.

**Figure 1:**
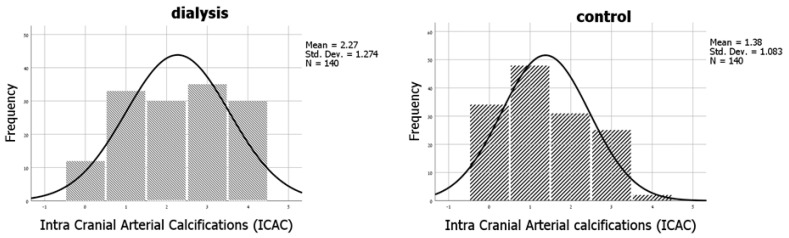
Distribution of intra-cranial calcifications among the study population.

The distribution of ICAC in the study population is shown in Figure 1. Mean ICAC was significantly higher in HD patients than in controls, 2.3 ± 1.3 vs. 1.4 ± 1.1, respectively (*p* < 0.01, Figure 1). A total of 24.3% of the control group did not have any calcifications on CT, as compared with only 8.6% in the dialysis group.

Comparison of the distribution of intracranial calcifications in dialysis versus the control group revealed a significantly different distribution, *p* < 0.01. Mean intra-cranial calcifications were 2.27 ± 1.27 in dialysis patients vs. 1.38 ± 1.08 in the control group, *p* < 0.01.

There was a trend toward increased prevalence of chronic Warfarin treatment with increasing severity of ICAC in the entire study population (*p* = 0.06), as well as in the subgroup of HD patients. Chronic Warfarin use among dialysis patients was higher in patients with ICAC = 1 or 2 (12.7%), as compared with those with ICAC = 0 (4%), *p* = 0.06, OR 3.5 (0.89–13.7). Mean ICAC was 2.64 ± 1.1 in dialysis patients treated with Warfarin and 2.25 ± 1.3 in dialysis patients that were not treated using Warfarin (*p* = 0.336).

**Figure 2:**
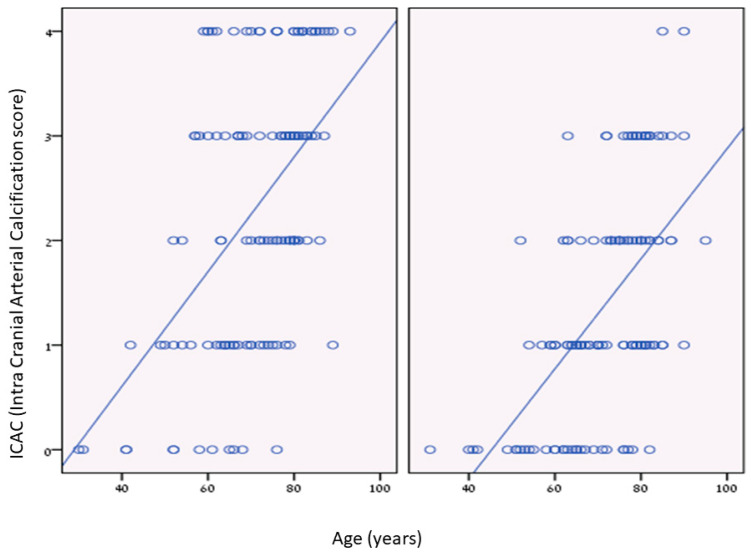
Intra Cranial Arterial calcifications (ICAC) as a function of age. (**Left**): correlation between age and intracranial calcifications (ICAC) score in dialysis patients; (**Right**): control patients. *p* < 0.01.

We found significant positive correlations between age and ICAC among dialysis patients and the control group. The dialysis group had more ICAC at younger ages than the control group (Figure 2).

Dialysis vintage did not correlate significantly with ICAC.

Serum phosphorus levels were positively correlated to ICAC in the dialysis group (mean serum Phosphorus 5.5 ± 1.9 mg/dL, *p* = 0.02) but not in the control group (mean serum Phosphorus 3.3 ± 0.6 mg/dL, *p* = 0.929). Calcium and PTH levels did not correlate with ICAC in either group (mean total calcium level 8.4 ± 0.9 mg/dL in dialysis and 9.3 ± 0.8 in the control group, *p* = 0.852, mean PTH level 309 ± 190 pg/mL in dialysis patients, *p* = 0.175). Albumin and CRP were correlated with ICAC in the dialysis group (*p* = 0.001, 0.007, respectively; albumin was inversely correlated with ICAC).

**Figure 3:**
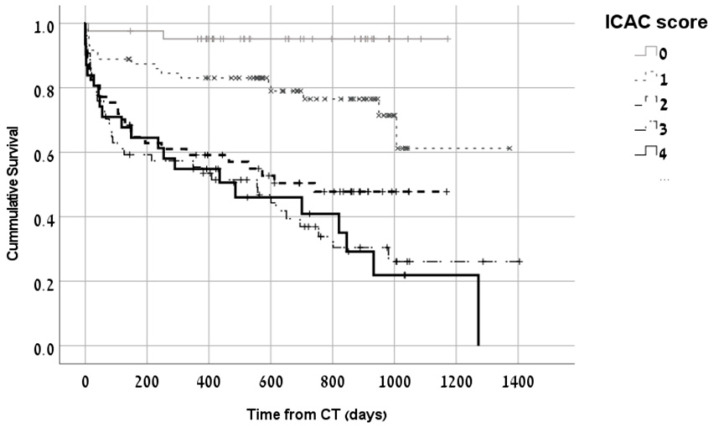
Kaplan–Meier survival curves of dialysis patients according to ICAC. Overall mortality over time was significantly higher for hemodialysis patients with higher ICAC scores, *p* < 0.01.

Mortality rates over time were higher among dialysis patients with higher ICAC. This remained significant even after adjusting for age and comorbidities. The survival curve of dialysis patients according to ICAC score is shown in Figure 3. The multivariate analysis model for predictors of 1-year mortality showed a significantly higher risk for mortality with increased ICAC (Table 2). Heart failure and ischemic heart disease were also significant predictors of 1-year mortality in the multivariate model.

## 4. Discussion

ICAC is easily identifiable in simple neuroimaging, such as non-contrast brain CT, which is often performed among dialysis patients. The presence and the severity of vascular calcification can be easily assessed for each patient and may serve as a cardiovascular marker [13]. In this study, we demonstrated that HD patients had a higher prevalence and severity of ICAC compared to a control group. These vascular calcifications also predicted mortality in dialysis patients and were significantly associated with their inflammatory and nutritional status.

More than 90% of hemodialysis patients in our study had some degree of ICAC. As previously described [7,10], ICAC was prevalent among our end-stage kidney disease patients probably since CKD and its associated comorbidities, including mineral bone disorder, are important risk factors for vascular calcifications.

The prevalence of risk factors such as age, diabetes mellitus, and atherosclerotic disease itself, manifested as ischemic heart disease and peripheral vascular disease, increased as the ICAC score increased. ICAC is arterial calcifications that are assumed to be an integral part of active atherosclerosis and were expected to be associated with these variables [6,7,8].

Although ICAC was higher as the Calcium-phosphorus product increased, we did not find any significant difference in serum levels of total calcium or phosphorus binder medications and ICAC score. There was a positive correlation between ICAC and phosphorus. Although calcium and phosphorus levels are important for the initiation and progression of calcifications, it is well-known that calcification is not a passive deposition of calcium and phosphate but a complex process resulting from many additional variables, and metabolic insults of diabetes, uremia, oxidative stress, and inflammation [10,14,15].

On correlation analysis, we found a significant association between the inflammatory marker CRP and a decrease in albumin as the ICAC score increased. The contribution of inflammation and malnutrition to vascular calcification was described by Choi et al. [14], probably a result of induction of osteogenic phenotype of vascular smooth muscle cells and stimulation of osteoblastic genes by inflammatory cytokines. Systemic inflammation, in both diabetic and non-diabetic environments, aggravates endothelial dysfunction and the atherosclerosis process [16,17,18,19].

Active inhibitors of vascular calcification are often down-regulated in patients with ESRD. One of these inhibitors is matrix Gla protein (MGP), a transcription factor that strongly inhibits vascular calcification and requires the activity of a vitamin K-dependent enzyme for carboxylation. Warfarin, a vitamin K antagonist, is assumed to promote vascular calcification via this mechanism [10,20]. In our cohort, 8% of HD patients were treated with Warfarin, and an increasing prevalence of Warfarin use was observed with higher ICAC scores. This increase is interesting and may be related to vitamin K deficiency associated with chronic use of Warfarin. The use of vitamin K antagonists for more than 3 months in adults was associated with a greater burden of intra-cranial calcifications among the elderly population [21].

Vascular calcification can occur in both the intima and media layers of blood vessels, while medial calcification is considered to be more common in patients with CKD, diabetes mellitus, and aging. Medial calcification is associated with increased aortic pulse wave velocity and arterial stiffness [10]. These hemodynamic changes may reduce myocardial perfusion during diastole, especially in the presence of coronary plaques, and lead to increased cardiovascular morbidity and mortality. This explains the increasing mortality as ICAC scores increased, which was reported in other studies, as well [7,8,10,15,22].

The interplay between blood pressure and dialysis is complex and has been extensively investigated. Dialysis patients may have high blood pressure as a result of volume overload, and as seen in our results, hypertension is prevalent among dialysis patients. The dialysis process itself may reduce blood pressure, leading to intradialytic hypotension, which may result in reduced brain blood flow, dialysis-related circulatory stress, regional ischemia, and finally, brain injury. Recently, Intradialytic magnetic resonance imaging (MRI) and spectroscopy demonstrated imaging changes characteristic of cytotoxic edema and ischemic injury [23]. These ischemic intradialytic changes in brain tissue occurred during a single dialysis session, and the coexistence of intracranial vascular calcifications and repeated dialysis sessions with its hemodynamics changes emphasize the importance of additional studies in this field.

In the current study, dialysis vintage did not correlate significantly with ICAC score on brain CT of dialysis patients. This finding is not consistent with other studies and may reflect the multifactorial nature of vascular calcifications and the limitations of the in vivo retrospective design of our study [4,6,7,24]. As mentioned above, vascular calcification is a complex process in disrupted mineral bone disorder, and atherosclerosis initiates a calcification cascade. In our study, ICAC was assessed at only one-time point and by brain CT. As shown by Shroff et al., the calcification process may rapidly progress on dialysis, with a dialysis vintage of even 2 months sufficient to induce histologically overt calcification [24].

The current study had some limitations. First, the study was based on a retrospective, single-center cohort; there might be a selection bias. Second, we did not use the gold standard method for diagnosing brain pathology—magnetic resonance imaging (MRI) because its use is less available in our institution, and we used data from available imaging that was performed to patients according to clinical indications as their physicians decided. The reason for performing the imaging was not available for all patients and was not adjusted between the groups. We do not have data about the FGF23-Klotho axis or specific mineral bone markers that are important players in vascular calcifications.

However, this study has several important clinical implications. We found that intracranial calcifications are very prevalent and severe among dialysis patients and are strongly associated with mortality. The finding of an available and inexpensive prognostic marker, such as ICAC, can help physicians stratify their patients’ risk in order to focus on different treatment strategies for higher-risk patients.

## 5. Conclusions

In conclusion, ICAC is highly prevalent and extensive among dialysis patients. Vascular calcifications do not simply result from passive depositions of calcium and phosphate but rather from complex, active processes involving inflammation and structural changes in blood vessels and hemodynamic changes. These result in increased mortality as the calcification score increases.

## Figures and Tables

**Table 1:** Baseline characteristics of patients according to ICAC score (N = 280).

Characteristic	ICAC Score 0 (n = 46)	ICAC Score 1,2 (n = 142)	ICAC Score 3,4 (n = 92)	*p*-Value *
Age, years	57 ± 14	72 ± 10	77 ± 9	<0.01
Male sex	27 (58.7)	90 (63.4)	53 (57.6)	0.65
Dialysis patients	12 (26.1)	63 (44.4)	65 (70.7)	<0.01
Dialysis vintage (months)	30 ± 70	10 ± 13	24 ± 26	0.148
Comorbidities				
Ischemic heart disease	5 (10.9)	37 (26.1)	40 (44.4)	<0.01
Heart failure	3 (6.5)	21 (14.8)	32 (35.2)	<0.01
Atrial Fibrillation	3 (6.5)	20 (14.1)	23 (25.3)	0.01
Prior stroke	10 (21.7)	38 (26.8)	31 (34.1)	0.27
Diabetes mellitus	10 (21.7)	58 (40.8)	59 (64.8)	<0.01
Peripheral vascular disease	1 (2.2)	10 (7.0)	13 (14.3)	0.04
Hypertension	22 (47.8)	108 (76.1)	76 (83.5)	<0.01
Chronic medications				
Aspirin	16 (34.8)	72 (50.7)	57 (63.3)	<0.01
Warfarin	1 (2.2)	5 (3.5)	9 (10.0)	0.06
Statins	16 (34.8)	66 (46.5)	47 (52.2)	0.16
Calcium Carbonate	6 (13.0)	24 (16.9)	20 (22.2)	0.38
Non-calcium-based phosphorus binders	3 (6.5)	10 (7.0)	13 (14.1)	0.15
Beta Blockers	10 (21.7)	64 (45.1)	48 (53.3)	<0.01
ACEI/ARB	8 (17.4)	42 (29.6)	32 (35.6)	0.09
Calcium channel blockers	10 (21.7)	40 (28.2)	35 (38.9)	0.08
Laboratory				
Hemoglobin (g/dL)	13.0 ± 1.8	12.0 ± 2.0	10.7 ± 2.0	<0.01
Albumin (gram/dL)	3.3 ± 0.7	3.4 ± 0.6	3.4 ± 0.6	0.6
Creatinine (mg/dL)	3.8 ± 12	2.9 ± 2.5	4.1 ± 2.6	0.2
Total calcium (mg/dL)	8.8 ± 0.9	8.9 ± 0.9	8.5 ± 0.8	0.04
Phosphorus (mg/dL)	4.1 ± 1.7	4.5 ± 2	5.2 ± 1.6	0.005
Calcium phosphorus product	35 ± 12	39 ± 16	44 ± 13	0.006
PTH (pg/mL)	268± 251	308 ± 187	314 ± 178	0.8
C reactive protein (mg/dL)	6.3 ± 9	6.6 ± 10	4.8 ± 6.7	0.3

ACEI, angiotensin-converting enzyme inhibitor; ARB, angiotensin II receptor blockers; PTH, parathyroid hormone. * Presented *p*-values represent the difference between the 3 ICAC categories.

**Table 2:** Multivariate analysis model for predictors of 1-year mortality among HD patients.

Coefficients	Odds Ratio	95% Confidence Interval		*p* Value
		Lower	Upper	
ICAC = 0	Reference			
ICAC = 1	3	0.6	14.7	0.2
ICAC = 2	9.3	2	44	0.005
ICAC = 3	8.4	1.8	40	0.007
ICAC = 4	8.2	1.6	43	0.01
Hypertension	1.4	0.6	3.3	0.4
Diabetes mellitus	1.0	0.5	2	0.9
Atrial fibrillation	0.7	0.3	1.6	0.4
Congestive heart failure	2.4	1.2	4.9	0.02
Ischemic heart disease	2.4	1.3	4.5	0.006
Peripheral vascular disease	1.6	0.6	4.3	0.3

Intra Cranial Calcification, ICAC.

## Data Availability

The data underlying this article will be shared on reasonable request to the corresponding author.
